# Bronchoscopic biological sealing of a bronchopleural fistula in post-tuberculous lung disease: a case report

**DOI:** 10.3389/fmed.2026.1987367

**Published:** 2026-10-14

**Authors:** Luoqi Yang, Kai Sun, Heng Zou, Hongwu Wang, Lu Wang

**Affiliations:** 1 Respiratory Disease Center, Dongzhimen Hospital, Beijing University of Chinese Medicine, Beijing, China; 2 Beijing University of Chinese Medicine, Beijing, China

**Keywords:** autologous blood, biological sealing, bronchopleural fistula, bronchoscopic intervention, post-tuberculous lung disease

## Abstract

**Background:**

Bronchopleural fistula (BPF) is an abnormal communication between the bronchial tree and pleural space that may result in persistent air leakage, impaired lung re-expansion, and pleural infection. Management can be particularly challenging in patients with chronic structural lung and pleural abnormalities.

**Case presentation:**

A 59-year-old man with a history of recurrent pulmonary tuberculosis presented with persistent left-sided pleural disease, air leakage during pleural drainage, and incomplete lung re-expansion. Serial chest computed tomography (CT) raised suspicion of a bronchopleural fistula. Sputum testing for Mycobacterium tuberculosis was negative, whereas microbiological examination of pleural fluid supported the presence of a superimposed infected pleural space. Under general anesthesia, flexible bronchoscopy was performed through a rigid bronchoscope. Methylene blue was injected through the left pleural drainage tube, and light-blue fluid was observed emerging from multiple subsegments of the left upper lobe apical segment, directly demonstrating the bronchopleural communication. A bronchoscopic biological sealing procedure was subsequently performed using a thrombin–autologous blood–thrombin sandwich technique. Two additional bronchoscopic interventions using thrombin and human fibrinogen were subsequently performed for reinforcement. On the third intervention, repeat methylene blue testing showed only a small amount of blue fluid leakage, which was no longer apparent after additional local thrombin application. However, persistent pleural infection required subsequent thoracic surgical management, including pleural debridement in November and further surgical management of the residual pleural space in December 2024. The patient remained clinically stable during follow-up through August 2026.

**Conclusion:**

Bronchoscopic biological sealing with autologous blood and thrombin may be a minimally invasive treatment option for selected patients with BPF and persistent air leakage. Methylene blue testing can facilitate direct localization of the bronchopleural communication and guide targeted bronchoscopic sealing. This case also illustrates that control of bronchopleural communication and management of an infected pleural space are distinct but complementary therapeutic goals, emphasizing the importance of adequate drainage and multidisciplinary management.

## 1 Introduction

Bronchopleural fistula (BPF) is an abnormal communication between the bronchial tree and pleural space. Although it is most frequently associated with thoracic surgery, BPF may also occur secondary to destructive pulmonary infections, including tuberculosis. In patients with previous pulmonary tuberculosis, chronic structural lung and pleural abnormalities may provide a clinical background for BPF formation. BPF is uncommon but can lead to persistent air leakage, pneumothorax, chronic pleural infection, and progressive loss of pulmonary function. Previous reports have described a broad clinical spectrum, ranging from incidental radiological findings to severe pneumothorax (1).

Management of BPF generally requires treatment of the underlying disease together with control of the pleural space and closure of the fistulous communication. Surgical repair may provide definitive treatment in selected patients; however, bronchoscopic interventions have increasingly been used as minimally invasive alternatives or salvage strategies, particularly when conventional drainage is insufficient or surgery is not immediately pursued (2). Available bronchoscopic approaches include biological sealants, autologous blood, endobronchial valves, occlusion devices, airway stents, and other mechanical techniques (3).

Autologous blood patch pleurodesis has been used for persistent air leaks for several decades, with clinical studies supporting its feasibility in selected patients (4). Importantly, tuberculosis-associated persistent air leaks treated with autologous blood patch pleurodesis have also been reported, including two cases published in 2024 (5). Therefore, the significance of the present case is not the first use of autologous blood in a patient with a history of tuberculosis, but methylene-blue-guided bronchoscopic localization of the bronchopleural communication followed by targeted thrombin–autologous blood–thrombin biological sealing and repeated bronchoscopic reinforcement.

Here, we describe the diagnostic evaluation and multidisciplinary management of a patient with chronic post-tuberculous structural lung and pleural disease complicated by BPF and an infected pleural space, with particular emphasis on methylene-blue-guided bronchoscopic localization and biological sealing.

## 2 Case presentation

### 2.1 Patient information and clinical history

A 59-year-old man with a history of pulmonary tuberculosis was referred to our tertiary hospital because of persistent air leakage requiring continuous pleural drainage and ongoing chest discomfort.

The patient was first diagnosed with pulmonary tuberculosis in 1999. Pulmonary tuberculosis recurred in 2013 and was accompanied by a left-sided encapsulated pleural effusion. From August 2013 to May 2015, he received regular oral anti-tuberculosis treatment with isoniazid, rifampicin, and ethambutol.

In April 2024, the patient developed increased cough and sputum production, particularly when lying on his side. Chest CT demonstrated a left-sided loculated hydropneumothorax, reduced left lung volume, and patchy nodular opacities in the left upper lung. His symptoms did not improve after antibacterial and expectorant therapy. Sputum testing for Mycobacterium tuberculosis was negative, and recurrent active pulmonary tuberculosis was not considered clinically at that time.

Because of worsening symptoms and persistent pleural disease, CT-guided left intercostal drainage was performed on August 29, 2024. Air bubbles were observed during drainage, suggesting communication between the airway and pleural cavity. Follow-up chest CT on September 2, compared with that on August 28, showed enlargement of the cavitary lesion in the left upper lung, persistent left-sided loculated pleural effusion with gas after drainage, incomplete expansion of the left lung, and possible pleuritis; a bronchopleural fistula could not be excluded. The patient was treated with oral nemonoxacin malate and fudosteine.

Approximately 15 days after discharge, grayish turbid fluid was again observed in the thoracic drainage bottle. Bacterial culture of the pleural fluid subsequently yielded Pseudomonas aeruginosa. He was treated with oral faropenem and fudosteine. Despite continued pleural drainage and medical treatment, persistent air leakage developed and continuous drainage remained necessary. The patient continued to experience chest discomfort and persistent air leakage and was therefore referred for further bronchoscopic evaluation and intervention.

### 2.2 Diagnostic assessment

Serial CT examinations were performed at Beijing Chest Hospital.

On August 28, 2024, chest CT showed possible infectious lesions in the left lung and the apical segment of the right upper lobe. A left-sided encapsulated pleural effusion was present, accompanied by pleural thickening and calcification, and pleuritis was considered possible.

On September 2, 2024, follow-up CT after left-sided intercostal drainage showed enlargement of the cavitary lesion in the left upper lung compared with the August 28 examination. The left-sided encapsulated pleural effusion persisted and was accompanied by pleural gas after drainage. A bronchopleural fistula could not be excluded. Pleural thickening and calcification remained evident. Patchy consolidation in the left lower lobe had decreased compared with the previous examination, and partial atelectasis was considered possible.

On September 30, 2024, CT showed no substantial interval change compared with September 2. The cavitary lesion in the left upper lung persisted, together with left-sided encapsulated pleural effusion and pleural gas after drainage. A bronchopleural fistula remained a diagnostic consideration (Figure 1, Table 1).

**Figure 1:**
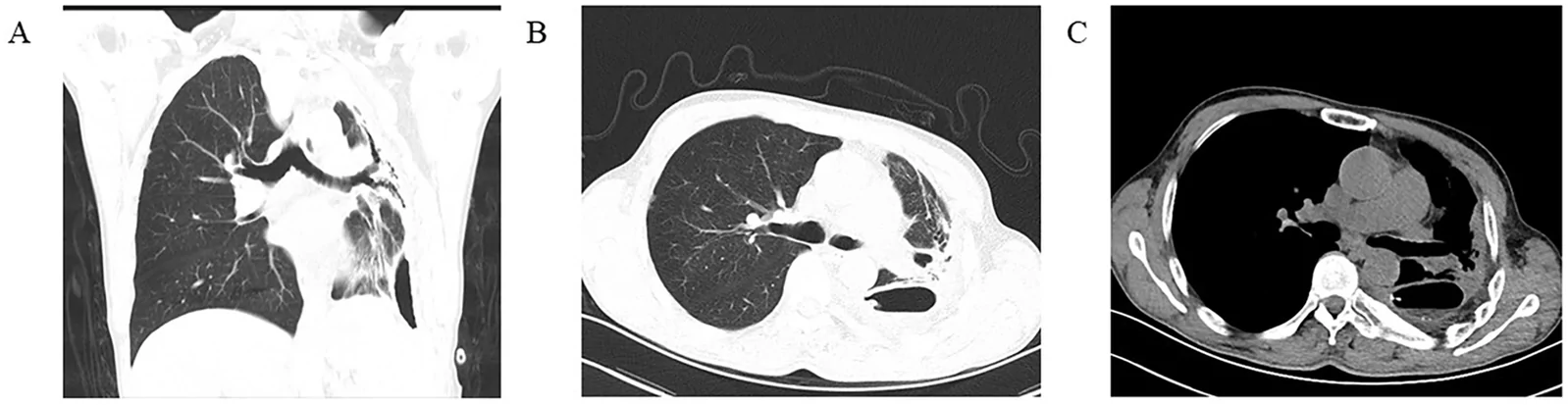
**(A–C)** pre-treatment chest CT scans showing a left upper lobe cavitary lesion and left-sided encapsulated pleural disease with pleural thickening and calcification.

**Table 1:** Timeline of the patient’s clinical course.

Date	Clinical event
1999	Pulmonary tuberculosis was initially diagnosed.
2013	Pulmonary tuberculosis recurred and was complicated by a left-sided encapsulated pleural effusion.
Aug 2013–May 2015	The patient received anti-tuberculosis treatment with isoniazid, rifampicin, and ethambutol.
April 2024	The patient developed worsening cough and sputum production, particularly in the lateral decubitus position. CT showed left-sided encapsulated pleural disease and abnormalities in the left upper lung.
Aug 28, 2024	Chest CT showed left-sided encapsulated pleural effusion with pleural thickening and calcification and abnormal lesions in the left lung.
Aug 29, 2024	CT-guided left pleural drainage was performed. Air bubbles were observed during drainage, raising suspicion of communication between the airway and pleural cavity.
Sep 2, 2024	Follow-up CT showed enlargement of the left upper lobe cavitary lesion, persistent encapsulated pleural effusion, and pleural gas; BPF could not be excluded.
Sep 12, 2024	Gray, turbid pleural drainage was observed. Pleural fluid culture grew *Pseudomonas aeruginosa*.
Sep 30, 2024	CT showed persistent left upper lobe cavitary lesion and left-sided encapsulated pleural disease with gas; BPF remained a diagnostic consideration.
Oct 17, 2024	Bronchoscopic methylene blue testing demonstrated communication between the pleural cavity and multiple subsegments of the apical segment of the left upper lobe.
Oct 22, 2024	First bronchoscopic biological sealing was performed using a thrombin–autologous blood–thrombin sequence.
Oct 29, 2024	Second bronchoscopic intervention was performed using thrombin–human fibrinogen–thrombin biological sealing.
Oct 30–Nov 1, 2024	Pleural drainage ceased, and catheter obstruction was suspected. CT-guided pleural drainage was re-established on November 1.
Nov 5, 2024	Third bronchoscopic reinforcement was performed. Repeat methylene blue testing showed markedly reduced leakage, with no obvious further leakage after additional local thrombin application.
Nov 8–11, 2024	Persistent purulent pleural drainage prompted thoracic surgical consultation and subsequent transfer for further surgical management.
Nov 18, 2024	Surgical pleural debridement was performed for persistent pleural infection.
Dec 2024	Further surgical management of the residual pleural space was performed.
Jan 2025	The pleural drainage catheter was removed.
Through Aug 2026	The patient remained clinically stable during follow-up. Long-term radiological follow-up was unavailable.

The combination of chronic post-tuberculous structural lung and pleural abnormalities, persistent left-sided pleural disease with gas, and continuous air leakage duringpleural drainage raised strong clinical suspicion of a bronchopleural fistula.

Before bronchoscopic intervention, laboratory testing showed mild anemia and inflammatory abnormalities, including hemoglobin 125 g/L, hematocrit 37.5%, mean platelet volume 6.3 fL, neutrophils 77.5%, lymphocytes 16.2%, eosinophils 0.0 × 10⁹/L, and C-reactive protein 10.77 mg/L. Albumin was 39.1 g/L, and blood glucose was 7.49 mmol/L. Pleural fluid culture obtained before referral yielded Pseudomonas aeruginosa. After admission to our hospital, metagenomic next-generation sequencing (mNGS) of purulent pleural fluid detected Pseudomonas aeruginosa, Achromobacter xylosoxidans, and Moraxella catarrhalis, as well as Aspergillus fumigatus; no viruses or parasites were detected. These microbiological findings supported the presence of infection within the pre-existing abnormal pleural space. During the current hospitalization, sputum testing for Mycobacterium tuberculosis was negative. Given the patient's previously completed course of anti-tuberculosis therapy and the absence of additional clinical evidence supporting tuberculosis recurrence, active pulmonary tuberculosis was not considered the primary cause of the current episode. To identify the fistulous communication, a bronchoscopy was performed on October 17, 2024. Methylene blue injection (10 mg/mL) was diluted by adding 1 mL to 500 mL of 0.9% sodium chloride solution, yielding a final concentration of approximately 0.020 mg/mL. Diluted methylene blue solution was injected through the left pleural drainage tube into the pleural cavity. Light-blue fluid was observed emerging from multiple subsegments of the left upper lobe apical segment, providing direct bronchoscopic evidence of communication between the pleural cavity and the bronchial tree. The apical segment of the left upper lobe represented the most clearly demonstrated site of communication.

The fistula diameter was not quantitatively measured because reliable measurement was not feasible during bronchoscopy.

### 2.3 Therapeutic intervention

Bronchoscopic biological sealing was performed under general anesthesia using a No. 12 rigid bronchoscope with a BF-P290 flexible bronchoscope (outer diameter, 4.2 mm). During the initial procedure on October 22, 2024, the central airways were patent, and no endobronchial neoplasm was identified. After removal of bronchial secretions, 100 mL of diluted methylene blue–sodium chloride solution was injected into the left pleural cavity through the indwelling pleural drainage tube. Light-blue fluid was observed emerging from multiple subsegments of the left upper lobe apical segment, confirming a bronchopleural communication. A bronchoscopic sandwich sealing technique was subsequently performed, consisting of 5 mL of thrombin preparation (100 IU/mL), 20 mL of autologous blood, and a further 5 mL of thrombin preparation (100 IU/mL), administered sequentially to the involved bronchial segments. Bronchospasm occurred during the procedure and was relieved after topical administration of 2% lidocaine. No active bleeding was observed during or after the procedure.

Follow-up chest CT on October 28, 2024, showed incomplete expansion of the left lung. Given the previously demonstrated bronchopleural communication and incomplete re-expansion of the left lung, a second bronchoscopic intervention was performed on October 29, 2024. After bronchoscopic inspection and removal of a small amount of white viscous secretions, 150 mL of the diluted methylene blue solution was instilled through the left pleural drainage catheter. Light-blue fluid was again observed emerging from the subsegmental bronchus of the left upper lobe apical segment, confirming persistence of the bronchopleural communication. A second biological sealing procedure was therefore performed using the sandwich technique with 3 mL of thrombin preparation (100 IU/mL), 20 mL of human fibrinogen (0.02 g/mL), and 3 mL of thrombin preparation (100 IU/mL). After withdrawal of the bronchoscope, gelatinous material was observed in the left main bronchus and was removed using biopsy forceps and CO_2_ cryotherapy. Following clearance, gelatinous material remained at the apical subsegmental bronchus, and the bronchial lumen appeared adequately sealed. Bronchospasm again occurred during the procedure and resolved after topical administration of 2% lidocaine. No active bleeding was observed during or after the procedure (Figure 2).

**Figure 2:**
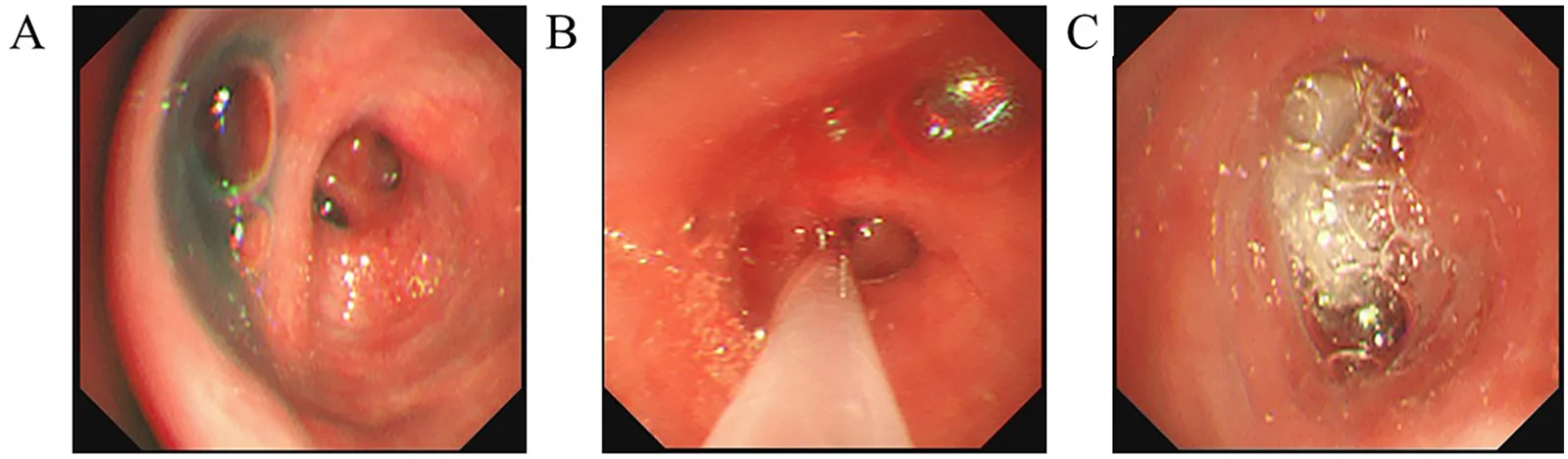
**(A)** bronchoscopic appearance before treatment. **(B)** Bronchoscopic appearance during thrombin–autologous blood–thrombin sandwich sealing. **(C)** Bronchoscopic appearance at subsequent re-examination after treatment.

Pleural drainage remained necessary during this treatment period. Daily drainage volumes ranged from 50 to 69 mL between October 26 and 29 but abruptly decreased to 0 mL on October 30. On October 31, attempted aspiration encountered marked negative pressure without recovery of purulent fluid, raising clinical suspicion of obstruction of the indwelling pleural drainage catheter. No fluid was obtained through the original catheter on November 1; the catheter was therefore removed and CT-guided pleural drainage was re-established, yielding 85 mL during the procedure. Drainage subsequently resumed (Table 2).

**Table 2:** Serial pleural drainage volumes and drainage-related events.

Date	Pleural drainage (mL)	Drainage-related event
Oct 26, 2024	50	Pleural drainage maintained
Oct 28, 2024	69	Pleural drainage maintained
Oct 29, 2024	50	Pleural drainage maintained
Oct 30, 2024	0	Abrupt cessation of drainage
Oct 31, 2024	0	Aspiration unsuccessful with marked negative pressure; catheter obstruction suspected
Nov 1, 2024	0*/85**	Original catheter yielded no drainage; CT-guided pleural drainage was re-established
Nov 4, 2024	100	Drainage resumed
Nov 5, 2024	75	Continued pleural drainage
Nov 6, 2024	135	Continued pleural drainage
Nov 7, 2024	60	Continued pleural drainage
Nov 8, 2024	10	Pleural fluid remained purulent
Nov 9, 2024	10	Continued pleural drainage
Nov 10, 2024	20	Continued pleural drainage

* No drainage through the original catheter.

** 85 mL was obtained during CT-guided re-establishment of pleural drainage.

A third bronchoscopic intervention was performed on November 5, 2024. The left upper lobe apical segment showed mild mucosal congestion and was prone to bleeding on contact, whereas the remaining left upper and lower lobe bronchi were patent without obvious endobronchial lesions. Following injection of 130 mL of diluted methylene blue–sodium chloride solution through the left pleural drainage tube, light-blue fluid was again observed emerging from the subsegmental bronchus of the left upper lobe apical segment. Further biological sealing was performed using repeated thrombin–fibrinogen–thrombin applications, including 2 mL of thrombin preparation (100 IU/mL), 3 mL of human fibrinogen (0.02 g/mL), and 2 mL of thrombin preparation (100 IU/mL), followed by multiple local injections of 0.5 mL of thrombin preparation, 1 mL of human fibrinogen, and 0.5 mL of thrombin preparation. After removal of scattered gelatinous material from the left main bronchus using biopsy forceps and CO_2_ cryotherapy, gelatinous material remained in place at the apical subsegmental bronchus, with the bronchial lumen appearing well sealed. A further 60 mL of diluted methylene blue–sodium chloride solution was then instilled through the pleural drainage tube. Only a small amount of light-blue fluid was observed to overflow initially, after which local spraying of thrombin preparation was performed. No obvious further leakage of blue fluid was observed. Bronchospasm occurred and was relieved with topical 2% lidocaine. Intraoperative bleeding was controlled with local application of a hemostatic thrombin preparation and argon plasma coagulation, with no significant active bleeding observed after the procedure.

### 2.4 Follow-up and outcomes

Following the initial bronchoscopic biological sealing on October 22, 2024, the patient's air leakage was markedly reduced but did not completely resolve. Two additional bronchoscopic reinforcement procedures were therefore performed on October 29 and November 5, 2024. During both procedures, methylene blue was again injected through the left pleural drainage tube and was observed emerging from the left upper lobe apical subsegment, confirming persistence of the bronchopleural communication. Additional thrombin–human fibrinogen–thrombin sealing was subsequently performed.

During the November 5 procedure, repeat methylene blue testing showed only a small amount of light-blue fluid emerging from the involved bronchial subsegment. After additional local application of thrombin, no obvious further leakage of blue fluid was observed bronchoscopically. These findings were accompanied by a substantial reduction in the clinically observed air leakage.

Pleural-space management remained necessary despite improvement in the bronchopleural air leak. Following replacement of the obstructed pleural drainage catheter on November 1, drainage resumed but remained purulent. On November 8, thoracic surgical consultation was recommended because of the persistent infected pleural space. Following consultation with thoracic surgeons at an outside hospital, transfer for surgical management was arranged on November 11.

Surgical pleural debridement was performed on November 18, 2024, for control of the persistent pleural infection. Further surgical management was performed on December 4, 2024, including pleural debridement and muscle-flap transposition for management of the residual pleural space.

Diagnostic-quality long-term radiological follow-up was unavailable. The pleural drainage catheter was removed in January 2025 after subsequent clinical management. The patient was followed clinically through August 2026, during which his condition remained stable, without reported recurrence of the major respiratory symptoms associated with the bronchopleural fistula. However, in the absence of diagnostic-quality long-term radiological follow-up was unavailable, the sustained clinical stability should not be interpreted as radiological confirmation of complete fistula closure or complete resolution of the underlying pleural disease.

## 3 Discussion

BPF is a clinically important complication because persistent communication between the bronchial tree and pleural space may maintain pneumothorax and air leakage and facilitate pleural infection. In patients with previous pulmonary tuberculosis, residual cavitation, fibrosis, pleural thickening, and chronic pleural inflammation may contribute to structural vulnerability and subsequent fistula formation. CT may demonstrate indirect findings such as persistent pneumothorax or hydropneumothorax, pleural air, failure of lung re-expansion, or a visible communication between the airway and pleural space (6, 7).

In the present case, the BPF was considered to occur in the setting of chronic post-tuberculous structural lung and pleural disease rather than active tuberculosis. Sputum testing for Mycobacterium tuberculosis was negative, whereas pleural fluid culture and mNGS provided evidence of a superimposed infected pleural space. These findings suggest that chronic post-tuberculous structural abnormalities complicated by pleural infection formed the clinical background for BPF development.

Diagnosis of BPF may be challenging, particularly when the fistulous opening is small or located at the subsegmental level. Bronchoscopy played an important role in establishing the anatomical communication. In this case, the fistulous communication was not simply inferred from CT findings. During flexible bronchoscopy, diluted methylene blue was injected through the pleural drainage tube. The appearance of blue fluid from the left upper lobe apical segment provided direct evidence of communication between the pleural cavity and bronchial tree. This approach also helped identify the bronchial region targeted for subsequent biological sealing.

Management of BPF depends on fistula size, location, underlying disease, pleural infection, respiratory reserve, and surgical candidacy. Surgical repair remains an important option, particularly for large or persistent fistulas and when adequate pleural debridement or source control is required. However, reoperation may be technically difficult in patients with extensive pleural adhesions, chronic infection, or poor general condition. Bronchoscopic treatment of BPF includes biological sealing materials, endobronchial valves, airway stents, and mechanical occlusion devices. Such approaches are particularly useful when surgical treatment is unsuitable or when a minimally invasive strategy is preferred. However, there is no universally accepted bronchoscopic technique for all BPFs, and treatment should be individualized according to fistula location, morphology, underlying disease, and pleural condition.

Autologous blood patch therapy has been used for persistent air leaks since the early 1990s. Its proposed mechanisms include mechanical obstruction by clot formation and induction of pleural inflammation leading to adhesion. Previous clinical studies have demonstrated its feasibility for persistent air leakage in selected patients (8). Importantly, tuberculosis-associated persistent air leaks treated with autologous blood patch pleurodesis have also been reported. Martinez et al. described two tuberculosis cases managed with autologous blood patch pleurodesis and endobronchial valves in 2024.

Repeated bronchoscopic biological sealing with autologous blood, thrombin, and fibrinogen was associated with progressively reduced methylene blue leakage, suggesting a reduction in bronchopleural communication. However, quantitative air-flow measurements were unavailable, and durable or complete fistula closure could not be confirmed. Importantly, control of bronchopleural communication and management of the infected pleural space represented distinct but complementary therapeutic goals (9). Pleural infection was present before bronchoscopic intervention and persisted despite improvement in air leakage. Although suspected catheter obstruction required CT-guided replacement, its cause remained undetermined. Persistent purulent drainage subsequently required surgical pleural debridement in November and further surgical management in December 2024. Thus, bronchoscopic biological sealing may serve as a minimally invasive component of multidisciplinary management but does not replace adequate drainage, infection control, or surgery when persistent pleural infection remains.

Several limitations should be acknowledged. First, this is a single case and cannot establish the efficacy or safety of the technique. Second, the fistula diameter was not quantitatively measured during bronchoscopy, preventing assessment of the relationship between fistula size and treatment response. Third, long-term follow-up was based primarily on clinical assessment because diagnostic-quality long-term radiological follow-up was unavailable, precluding objective confirmation of durable fistula closure or complete resolution of the underlying pleural disease. Finally, the patient subsequently underwent surgical treatment; therefore, the long-term clinical outcome cannot be attributed solely to bronchoscopic biological sealing.

## 4 Conclusion

Bronchoscopic biological sealing with autologous blood, thrombin, and fibrinogen may provide a minimally invasive approach for selected patients with BPF in the setting of chronic post-tuberculous structural lung and pleural disease. Methylene blue facilitated localization of the bronchopleural communication, while repeated biological sealing was associated with reduced bronchoscopically observed leakage. This case also highlights that control of bronchopleural communication does not replace adequate drainage, infection control, or surgical management when persistent pleural infection remains.

## 5 Patient perspective

The patient reported that persistent chest discomfort and prolonged drainage had substantially affected his daily life. He appreciated the minimally invasive nature of the bronchoscopic treatment and reported improvement in respiratory symptoms after the procedure. He understood that the intervention reduced the air leakage but that additional treatment was required because of subsequent pleural drainage problems.

## Data Availability

The original contributions presented in the study are included in the article/Supplementary Material, further inquiries can be directed to the corresponding author.
